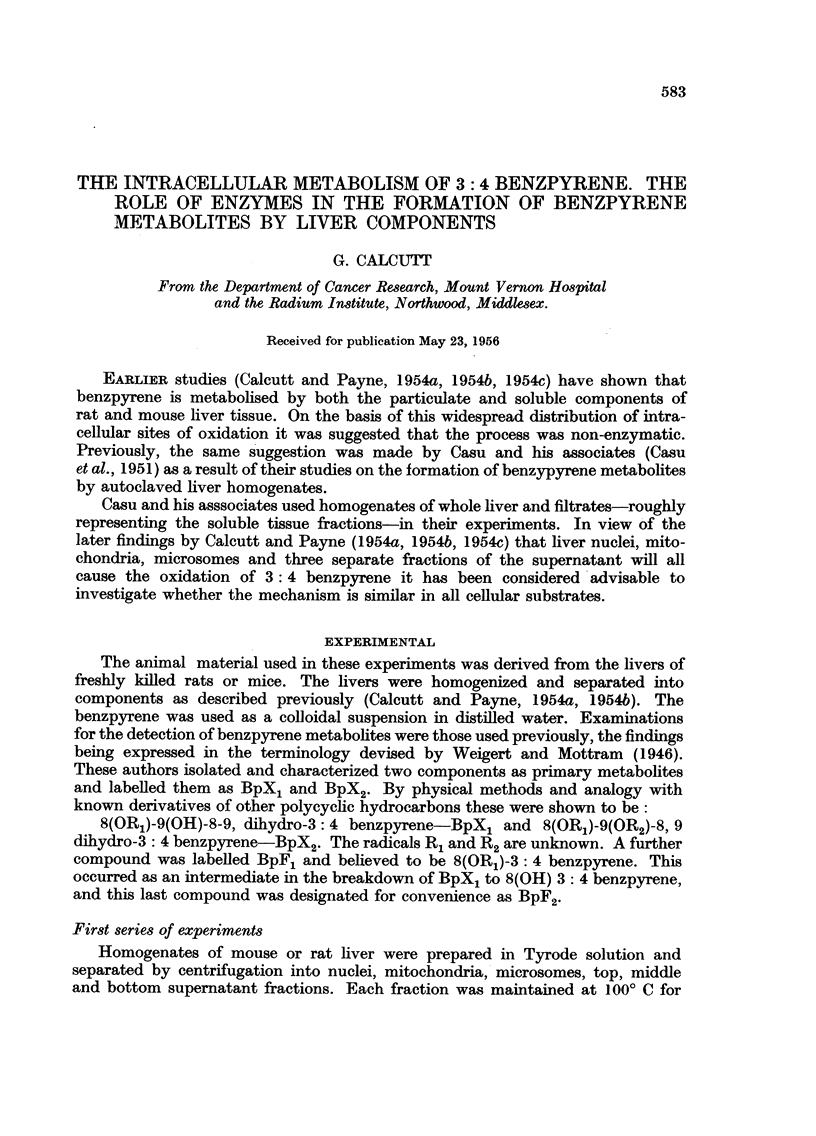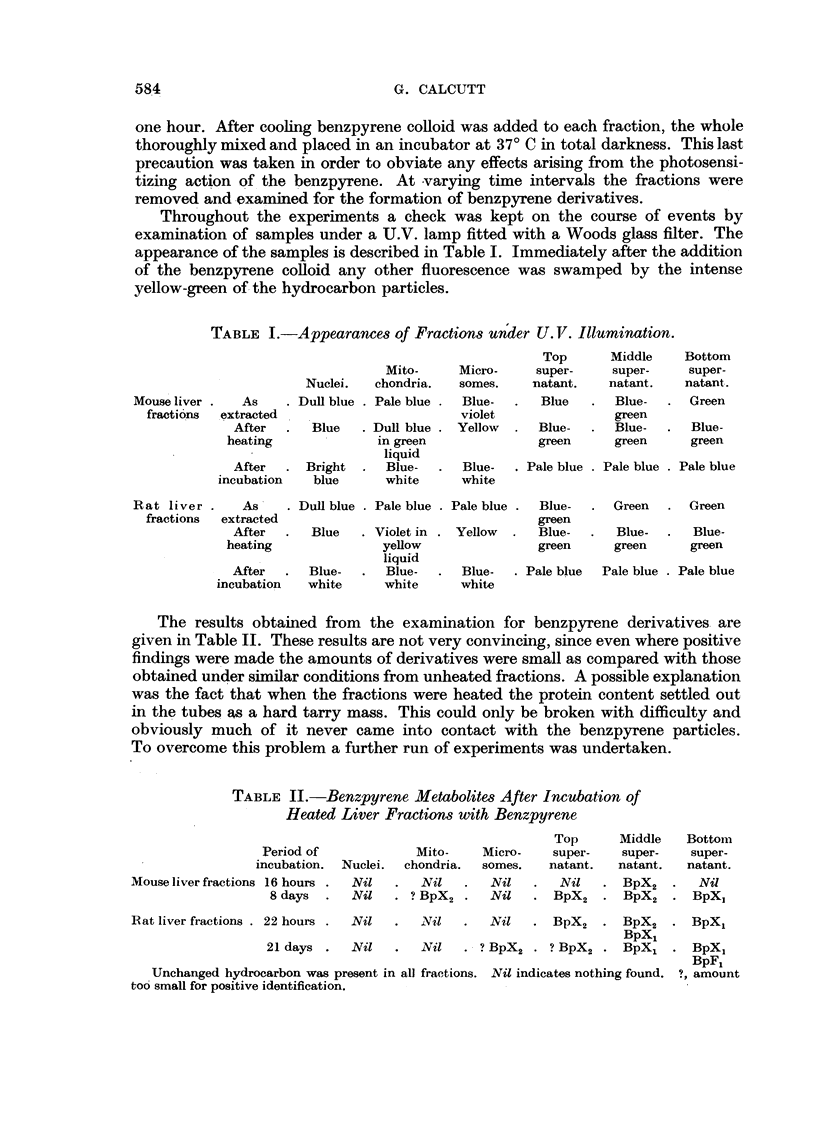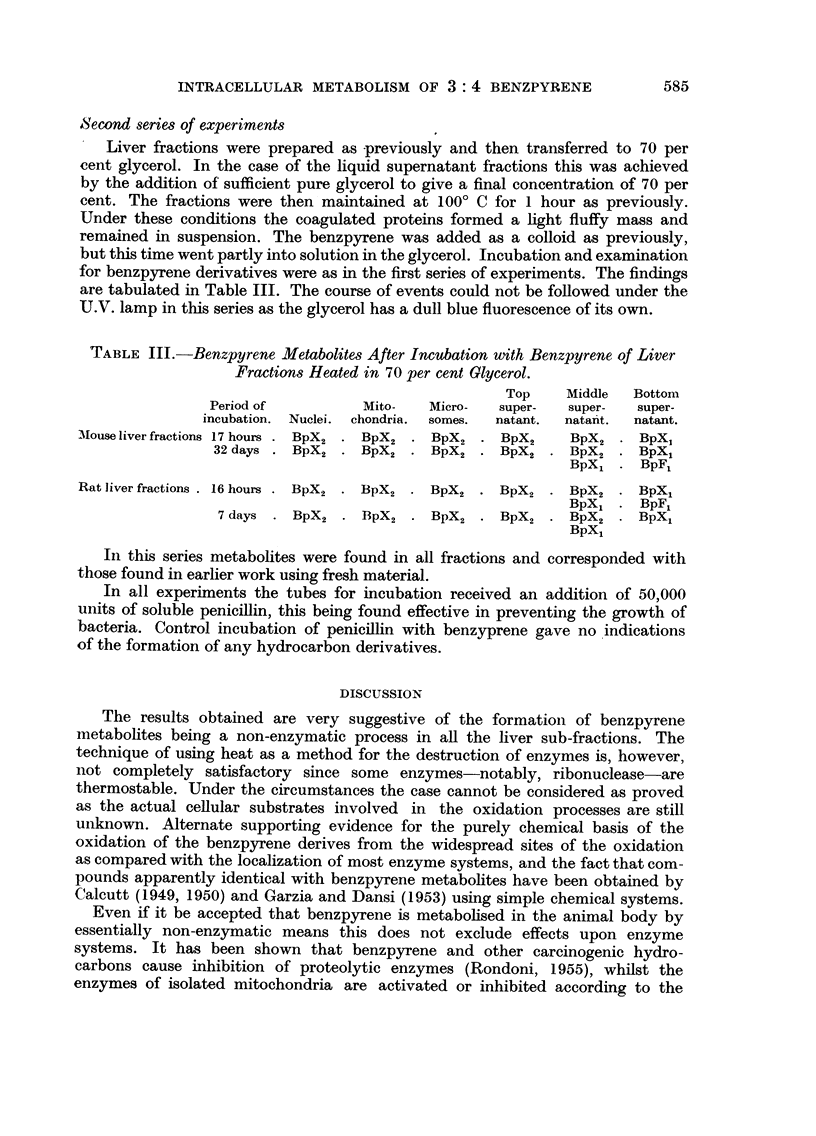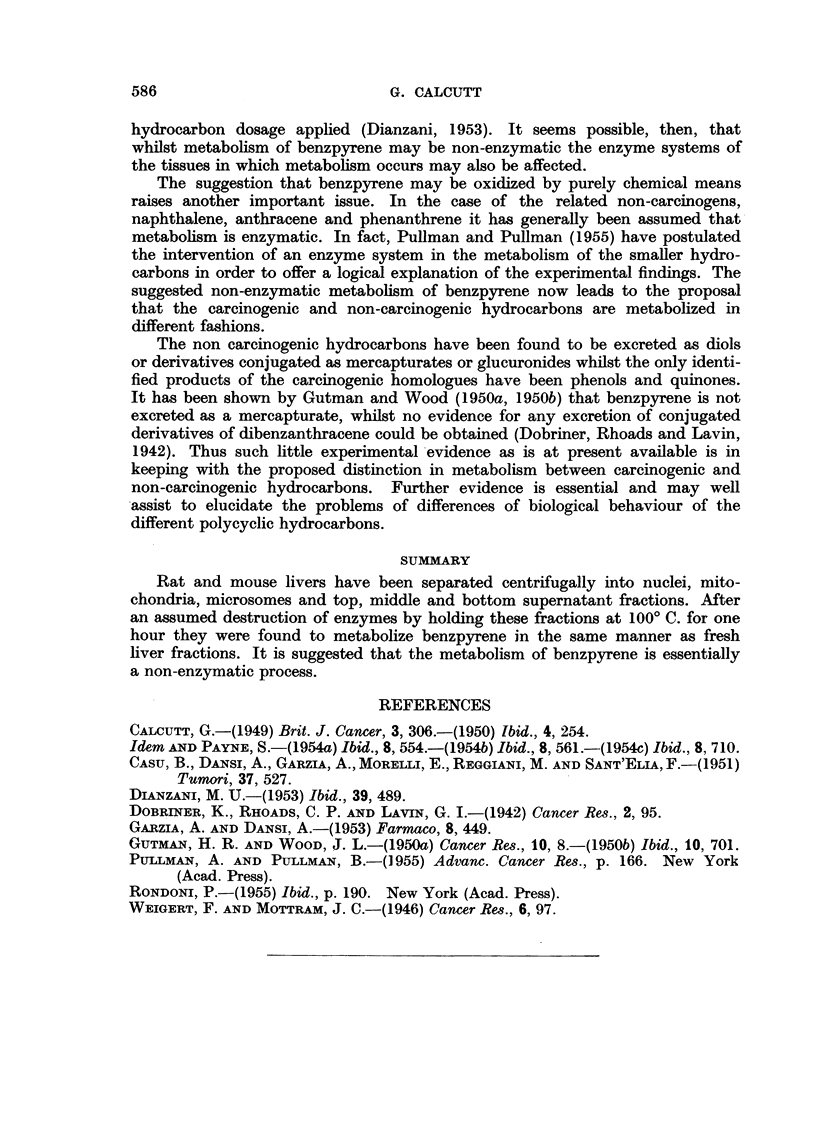# The Intracellular Metabolism of 3:4 Benzpyrene. The Role of Enzymes in the Formation of Benzpyrene Metabolites by Liver Components

**DOI:** 10.1038/bjc.1956.70

**Published:** 1956-09

**Authors:** G. Calcutt


### THE INTRACELLULAR METABOLISM OF 3 : 4 BENZPYRENE. THE ROLE OF ENZYMES IN THE FORMATION OF BENZPYRENE METABOLITES BY LIVER COMPONENTS

G. CALCUTT

*From the Department of Cancer Research, Mount Vernon Hospital and the Radium Institute, Northwood, Middlesex.*

Received for publication May 23, 1956

EARLIER studies (Calcutt and Payne, 1954*a*, 1954*b*, 1954*c*) have shown that benzpyrene is metabolised by both the particulate and soluble components of rat and mouse liver tissue. On the basis of this widespread distribution of intracellular sites of oxidation it was suggested that the process was non-enzymatic. Previously, the same suggestion was made by Casu and his associates (Casu *et al.*, 1951) as a result of their studies on the formation of benzypyrene metabolites by autoclaved liver homogenates.

Casu and his asssociates used homogenates of whole liver and filtrates—roughly representing the soluble tissue fractions—in their experiments. In view of the later findings by Calcutt and Payne (1954*a*, 1954*b*, 1954*c*) that liver nuclei, mitochondria, microsomes and three separate fractions of the supernatant will all cause the oxidation of 3 : 4 benzpyrene it has been considered advisable to investigate whether the mechanism is similar in all cellular substrates.

### EXPERIMENTAL

The animal material used in these experiments was derived from the livers of freshly killed rats or mice. The livers were homogenized and separated into components as described previously (Calcutt and Payne, 1954*a*, 1954*b*). The benzpyrene was used as a colloidal suspension in distilled water. Examinations for the detection of benzpyrene metabolites were those used previously, the findings being expressed in the terminology devised by Weigert and Mottram (1946). These authors isolated and characterized two components as primary metabolites and labelled them as $BpX_1$ and $BpX_2$. By physical methods and analogy with known derivatives of other polycyclic hydrocarbons these were shown to be :

$8(OR_1)$-9(OH)-8-9, dihydro-3 : 4 benzpyrene—$BpX_1$ and $8(OR_1)$-$9(OR_2)$-8, 9 dihydro-3 : 4 benzpyrene—$BpX_2$. The radicals $R_1$ and $R_2$ are unknown. A further compound was labelled $BpF_1$ and believed to be $8(OR_1)$-3 : 4 benzpyrene. This occurred as an intermediate in the breakdown of $BpX_1$ to 8(OH) 3 : 4 benzpyrene, and this last compound was designated for convenience as $BpF_2$.

### *First series of experiments*

Homogenates of mouse or rat liver were prepared in Tyrode solution and separated by centrifugation into nuclei, mitochondria, microsomes, top, middle and bottom supernatant fractions. Each fraction was maintained at 100° C for one hour. After cooling benzpyrene colloid was added to each fraction, the whole thoroughly mixed and placed in an incubator at 37° C in total darkness. This last precaution was taken in order to obviate any effects arising from the photosensitizing action of the benzpyrene. At varying time intervals the fractions were removed and examined for the formation of benzpyrene derivatives.

Throughout the experiments a check was kept on the course of events by examination of samples under a U.V. lamp fitted with a Woods glass filter. The appearance of the samples is described in Table I. Immediately after the addition of the benzpyrene colloid any other fluorescence was swamped by the intense yellow-green of the hydrocarbon particles.

TABLE I.—*Appearances of Fractions under U.V. Illumination.*

| | | Nuclei. | Mito-chondria. | Micro-somes. | Top super-natant. | Middle super-natant. | Bottom super-natant. |
|---|---|---|---|---|---|---|---|
| Mouse liver fractions | As extracted | Dull blue | Pale blue | Blue-violet | Blue | Blue-green | Green |
| | After heating | Blue | Dull blue in green liquid | Yellow | Blue-green | Blue-green | Blue-green |
| | After incubation | Bright blue | Blue-white | Blue-white | Pale blue | Pale blue | Pale blue |
| Rat liver fractions | As extracted | Dull blue | Pale blue | Pale blue | Blue-green | Green | Green |
| | After heating | Blue | Violet in yellow liquid | Yellow | Blue-green | Blue-green | Blue-green |
| | After incubation | Blue-white | Blue-white | Blue-white | Pale blue | Pale blue | Pale blue |

The results obtained from the examination for benzpyrene derivatives are given in Table II. These results are not very convincing, since even where positive findings were made the amounts of derivatives were small as compared with those obtained under similar conditions from unheated fractions. A possible explanation was the fact that when the fractions were heated the protein content settled out in the tubes as a hard tarry mass. This could only be broken with difficulty and obviously much of it never came into contact with the benzpyrene particles. To overcome this problem a further run of experiments was undertaken.

TABLE II.—*Benzpyrene Metabolites After Incubation of Heated Liver Fractions with Benzpyrene*

| | Period of incubation. | Nuclei. | Mito-chondria. | Micro-somes. | Top super-natant. | Middle super-natant. | Bottom super-natant. |
|---|---|---|---|---|---|---|---|
| Mouse liver fractions | 16 hours | *Nil* | *Nil* | *Nil* | *Nil* | $BpX_2$ | *Nil* |
| | 8 days | *Nil* | ? $BpX_2$ | *Nil* | $BpX_2$ | $BpX_2$ | $BpX_1$ |
| Rat liver fractions | 22 hours | *Nil* | *Nil* | *Nil* | $BpX_2$ | $BpX_2$ $BpX_1$ | $BpX_1$ |
| | 21 days | *Nil* | *Nil* | ? $BpX_2$ | ? $BpX_2$ | $BpX_1$ | $BpX_1$ $BpF_1$ |

Unchanged hydrocarbon was present in all fractions. *Nil* indicates nothing found. ?, amount too small for positive identification.

*Second series of experiments*

Liver fractions were prepared as previously and then transferred to 70 per cent glycerol. In the case of the liquid supernatant fractions this was achieved by the addition of sufficient pure glycerol to give a final concentration of 70 per cent. The fractions were then maintained at 100° C for 1 hour as previously. Under these conditions the coagulated proteins formed a light fluffy mass and remained in suspension. The benzpyrene was added as a colloid as previously, but this time went partly into solution in the glycerol. Incubation and examination for benzpyrene derivatives were as in the first series of experiments. The findings are tabulated in Table III. The course of events could not be followed under the U.V. lamp in this series as the glycerol has a dull blue fluorescence of its own.

TABLE III.—*Benzpyrene Metabolites After Incubation with Benzpyrene of Liver Fractions Heated in* 70 *per cent Glycerol.*

| | Period of incubation. | Nuclei. | Mito-chondria. | Micro-somes. | Top super-natant. | Middle super-natant. | Bottom super-natant. |
|---|---|---|---|---|---|---|---|
| Mouse liver fractions | 17 hours | $BpX_2$ | $BpX_2$ | $BpX_2$ | $BpX_2$ | $BpX_2$ | $BpX_1$ |
| | 32 days | $BpX_2$ | $BpX_2$ | $BpX_2$ | $BpX_2$ | $BpX_2$ | $BpX_1$ |
| | | | | | | $BpX_1$ | $BpF_1$ |
| Rat liver fractions | 16 hours | $BpX_2$ | $BpX_2$ | $BpX_2$ | $BpX_2$ | $BpX_2$ | $BpX_1$ |
| | | | | | | $BpX_1$ | $BpF_1$ |
| | 7 days | $BpX_2$ | $BpX_2$ | $BpX_2$ | $BpX_2$ | $BpX_2$ | $BpX_1$ |
| | | | | | | $BpX_1$ | |

In this series metabolites were found in all fractions and corresponded with those found in earlier work using fresh material.

In all experiments the tubes for incubation received an addition of 50,000 units of soluble penicillin, this being found effective in preventing the growth of bacteria. Control incubation of penicillin with benzyprene gave no indications of the formation of any hydrocarbon derivatives.

### DISCUSSION

The results obtained are very suggestive of the formation of benzpyrene metabolites being a non-enzymatic process in all the liver sub-fractions. The technique of using heat as a method for the destruction of enzymes is, however, not completely satisfactory since some enzymes—notably, ribonuclease—are thermostable. Under the circumstances the case cannot be considered as proved as the actual cellular substrates involved in the oxidation processes are still unknown. Alternate supporting evidence for the purely chemical basis of the oxidation of the benzpyrene derives from the widespread sites of the oxidation as compared with the localization of most enzyme systems, and the fact that compounds apparently identical with benzpyrene metabolites have been obtained by Calcutt (1949, 1950) and Garzia and Dansi (1953) using simple chemical systems.

Even if it be accepted that benzpyrene is metabolised in the animal body by essentially non-enzymatic means this does not exclude effects upon enzyme systems. It has been shown that benzpyrene and other carcinogenic hydrocarbons cause inhibition of proteolytic enzymes (Rondoni, 1955), whilst the enzymes of isolated mitochondria are activated or inhibited according to the hydrocarbon dosage applied (Dianzani, 1953). It seems possible, then, that whilst metabolism of benzpyrene may be non-enzymatic the enzyme systems of the tissues in which metabolism occurs may also be affected.

The suggestion that benzpyrene may be oxidized by purely chemical means raises another important issue. In the case of the related non-carcinogens, naphthalene, anthracene and phenanthrene it has generally been assumed that metabolism is enzymatic. In fact, Pullman and Pullman (1955) have postulated the intervention of an enzyme system in the metabolism of the smaller hydrocarbons in order to offer a logical explanation of the experimental findings. The suggested non-enzymatic metabolism of benzpyrene now leads to the proposal that the carcinogenic and non-carcinogenic hydrocarbons are metabolized in different fashions.

The non carcinogenic hydrocarbons have been found to be excreted as diols or derivatives conjugated as mercapturates or glucuronides whilst the only identified products of the carcinogenic homologues have been phenols and quinones. It has been shown by Gutman and Wood (1950*a*, 1950*b*) that benzpyrene is not excreted as a mercapturate, whilst no evidence for any excretion of conjugated derivatives of dibenzanthracene could be obtained (Dobriner, Rhoads and Lavin, 1942). Thus such little experimental evidence as is at present available is in keeping with the proposed distinction in metabolism between carcinogenic and non-carcinogenic hydrocarbons. Further evidence is essential and may well assist to elucidate the problems of differences of biological behaviour of the different polycyclic hydrocarbons.

### SUMMARY

Rat and mouse livers have been separated centrifugally into nuclei, mitochondria, microsomes and top, middle and bottom supernatant fractions. After an assumed destruction of enzymes by holding these fractions at 100° C. for one hour they were found to metabolize benzpyrene in the same manner as fresh liver fractions. It is suggested that the metabolism of benzpyrene is essentially a non-enzymatic process.

### REFERENCES

Calcutt, G.—(1949) *Brit. J. Cancer*, **3**, 306.—(1950) *Ibid.*, **4**, 254.

*Idem* and Payne, S.—(1954*a*) *Ibid.*, **8**, 554.—(1954*b*) *Ibid.*, **8**, 561.—(1954*c*) *Ibid.*, **8**, 710.

Casu, B., Dansi, A., Garzia, A., Morelli, E., Reggiani, M. and Sant'Elia, F.—(1951) *Tumori*, **37**, 527.

Dianzani, M. U.—(1953) *Ibid.*, **39**, 489.

Dobriner, K., Rhoads, C. P. and Lavin, G. I.—(1942) *Cancer Res.*, **2**, 95.

Garzia, A. and Dansi, A.—(1953) *Farmaco*, **8**, 449.

Gutman, H. R. and Wood, J. L.—(1950*a*) *Cancer Res.*, **10**, 8.—(1950*b*) *Ibid.*, **10**, 701.

Pullman, A. and Pullman, B.—(1955) *Advanc. Cancer Res.*, p. 166. New York (Acad. Press).

Rondoni, P.—(1955) *Ibid.*, p. 190. New York (Acad. Press).

Weigert, F. and Mottram, J. C.—(1946) *Cancer Res.*, **6**, 97.